# Gun Cotton in Blasting

**Published:** 1847-06

**Authors:** 


### SELECTIONS

FROM

### AMERICAN AND FOREIGN JOURNALS.

---

*Gun Cotton in Blasting.*—We take the following from a London paper, for which we are indebted to our Paris correspondent:

A series of trial experiments to test the strength of gun-cotton in blasting, as compared with the known powers of gunpowder, took place on Wednesday, in the Standedge tunnel, on the Huddersfield and Manchester Railway. A correspondent thus describes the proceedings and their results. The place selected for the experiments is in the midst of the great Yorkshire chain of hills, between Huddersfield and Manchester, where a tunnel is now constructing through huge masses of Millstone grit, and which, when completed, will be the longest tunnel in England. The rocks, which are situate at the depth of one hundred and fifty-five yards below the surface of the mountain, consist entirely of close grained quartz, of which some idea may be formed by the fact that in one hundred and ten yards of the tunnel now completed, not one fissure or joint is perceptible. Holes were drilled of 2 inches, 1⅛ inch, and 1¼ inch in diameter in the millstone grit, and the charges of gun-cotton varied from 1-6th to 1-3rd of the weight of powder ordinarily made use of in blasting. Cartridges containing the gun-cotton were dropped into the holes with the patent fuse attached, and the quantities varied from 10 ounces in the larger, to 6 ounces in the smaller tubes; the quantity of gunpowder employed for similar blast-drills, varying from 1 to 3 lbs. The shots in the tunnel were 12 in number, 10 being charged with gun-cotton, and No. 4 and 11 with gunpowder. The shots numbered 2 and 7 in the south heading and 12 in the north heading, were declared by Mr. Nicholson, the contractor, previous to their being fired, to be those on which the test of the strength of gun-cotton, compared with that of gunpowder, would be most certain, both as to charges and position. No. 2 was charged with 10 ounces of gun-cotton, and did wonderful execution, exceeding in everything the expectations of both the engineers and miners. No. 7 was charged with 1¼ ounce of gun-cotton, and performed the same work as 6 ounces of powder. The crowning experiments were No. 11 and 12 in the north heading. No. 12 was charged with 16 ounces gun-cotton against 3lb. 4oz. of gunpowder in No. 11, the shots exceeding anything the engineers or miners had before tested. The gun-cotton exploded first, and threw against the side of the heading, and broke into nine pieces, 220 cubic feet of rock, or about 15 tons weight. This shot, from its connection with No. 11, was supposed to have assisted it materially. No. 11 removed, to the distance of about one yard, 224 feet of rock, or about 16½ tons weight, but was broken into six pieces only. Thus showing the strength of one over the other. The following properties were established in these experiments: That the gun-cotton may be considered of three or four times the strength of gunpowder; that it is entirely free from smoke, a qualification of great importance in mining operations, as the miners can recommence work immediately after the blasts have been fired, which is not the case with gunpowder; that in keeping the cotton on the works, and its being handled by the men, there is not so much risk from explosion or loss from waste as with gunpowder; and, from the decreased size of the charge, the depth of the drill-hole may be diminished, and the power more concentrated: it also possesses all the qualities of the best gunpowder, and may be kept under water instead of in a magazine; it does not heat by keeping, and will explode at not less than 350.

